# Prévalence des complications de la corticothérapie chez les sujets ouest-africains consultant en rhumatologie

**DOI:** 10.11604/pamj.2015.21.304.5805

**Published:** 2015-08-26

**Authors:** Zavier Zomalheto, Hilaire Dossou-yovo, Fidèle Zossoungbo, Martin Avimadjè

**Affiliations:** 1 Service de Rhumatologie du Centre National Hospitalo-Universitaire Hubert Koutoukou Maga de Cotonou, Cotonou, Benin

**Keywords:** Corticoïdes, rhumatologie, complications, Cotonou, corticosteroids, rheumatology, complications, Cotonou

## Abstract

**Introduction:**

Les corticoïdes sont pourvoyeurs d'effets secondaires multiples responsables de leur mauvaise réputation. L'objectif de ce travail a été d’établir la prévalence des complications de la thérapie cortisonique chez les sujets ouest-africains souffrant d'affection rhumatologique au CNHU-HKM de Cotonou.

**Méthodes:**

Il s'agissait d'une étude rétrospective sur 5 ans portant sur des dossiers de patients de diverses nationalités reçus dans l'unité de rhumatologie du CNHU-HKM de Cotonou. Nous avons répertorié les complications liées à la prise des corticoïdes donnés dans le cadre des pathologies rhumatologiques. Les données ont été analysées grâce au logiciel EXCELL version 2013.

**Résultats:**

745 (17,1%) avaient reçu l'indication d'une corticothérapie. Ils étaient de nationalités diverses de l'Afrique de l'ouest dont 87,4% de béninois. L’âge moyen des patients était de 51,15±21,58 (10-73) ans. La sex ratio était 0,61. Il s'agissait d'une corticothérapie par voie générale dans 65,1%. Les complications étaient présentes chez 31,1% des patients toute voie confondue. Celles engendrées par les infiltrations étaient dominées par la décoloration cutanée en regard du site d'injection (40%) alors que la corticothérapie générale était responsable de la prise de poids dans plus d'un cas sur 2.

**Conclusion:**

Les complications de la thérapie cortisonique sont diverses et variées. La manipulation des dérivés cortisoniques doit se faire avec prudence en tenant compte du terrain et surtout de l’évaluation à chaque moment du rapport bénéfice-risque.

## Introduction

Malgré l'ancienneté de la corticothérapie, celle-ci demeure une thérapeutique extrêmement utilisée par voie générale et locale dans les spécialités cliniques. La corticothérapie systémique est utilisée en thérapeutique depuis 60 ans et a bouleversé le pronostic de la plupart des maladies inflammatoires. Par son efficacité et sa rapidité d'action, elle reste souvent le traitement de première intention de la plupart de ces maladies. On estime que 0,2 à 0,5% de la population générale reçoit une corticothérapie systémique prolongée au-delà de plus de trois mois [1–3]. En Angleterre, près de 1% de la population adulte et jusqu’à 2,5% de la population de plus de 70 ans prend une corticothérapie au long cours [1]. Aux États-Unis, une nouvelle corticothérapie orale est instituée chez dix millions de personnes par an [4]. Les corticoïdes affectent la synthèse des prostaglandines en bloquant deux voies de synthèse de l'acide arachidonique: la cyclo-oxygénase et la lipo-oxygénase. L'effet anti-inflammatoire et immunomodulateur des corticoïdes est donc particulièrement recherché en thérapeutique.Les corticoïdes sont cependant pourvoyeurs d'effets secondaires multiples responsables de leur mauvaise réputation [4, 5]. La fréquence des effets indésirables est fonction de nombreux facteurs: terrain (âge, antécédents pathologiques), posologie quotidienne, dose totale, durée du traitement, nature du corticoïde utilisé, voie et mode d'administration, maladie traitée, etc… La majorité des effets indésirables, pouvant survenir au cours d'une corticothérapie systémique, est prévisible car liée à l'effet pharmacologique. Dans ce cas, ils peuvent être prévenus par des mesures diététiques et/ou médicamenteuses. La fréquence de la plupart des effets indésirables attribués à une corticothérapie systémique prolongée a rarement été évaluée de façon systématique [2, 6]. Dans une étude prospective française portant sur 80 patients, Fardet et coll. ont montré que 71% des patients rapportent au moins un effet indésirable [7]. L'objectif de ce travail est d’établir la prévalence des complications de la thérapie cortisonique chez les sujets ouest-africains souffrant consultant en rhumatologie au CNHU-HKM de Cotonou.

## Méthodes

Il s'agit d'une étude rétrospective de janvier 2010 à Juillet 2014 portant sur des dossiers de patients reçus dans l'unité de rhumatologie du CNHU-HKM de Cotonou. Les patients inclus dans l’étude répondaient aux critères suivants: avoir consulté dans l'unité de rhumatologie pendant la période d’étude; avoir souffert d'une affection rhumatismale; avoir reçu une thérapeutique cortisonique; avoir présenté des effets secondaires en rapport avec la corticothérapie et notifiés dans le dossier médical. Les patients perdus de vu et irrégulièrement suivis n'ont pas été inclus dans l’étude. Les données recueillies à partir des dossiers et transcrites sur une fiche préétablie ont été analysées grâce aux logiciels épidata et SPSS17.0. Le test de chi2 a servi pour les comparaisons des données. La différence est significative pour une valeur de p < 0,05.

## Résultats

### Caractères socio-démographiques

Parmi 4361 patients consultés, 745 (17,1%) avaient reçu l'indication d'une corticothérapie et répondaient aux critères d'inclusion. L’âge moyen des patients était de 51,15±21,58 (10-73) ans et la sex ratio était 0,61. Les patients étaient de nationalités diverses de l'Afrique de l'ouest (87,4% de béninois, 6,5% de nigérians, 2,6% maliens, 1,1% togolais, 0,9% burkinabés, 0,8% d'ivoiriens, 0,7% sénégalais).

### Indications de la corticothérapie

**Figure 1:**
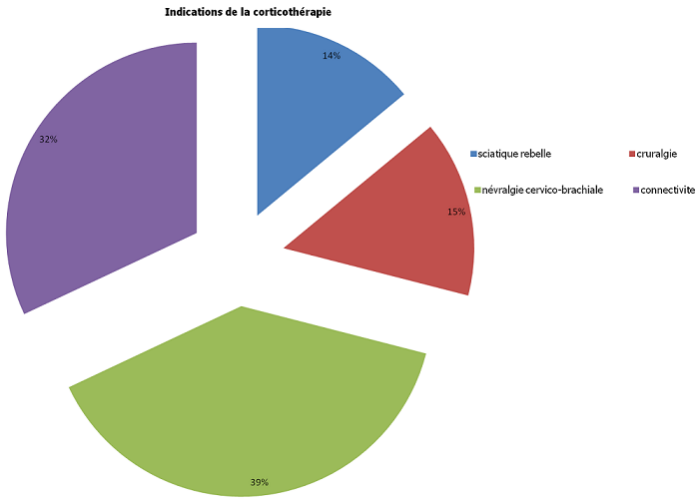
Pathologies ayant motivé la corticothérapie générale dans le service de rhumatologie du CNHU-HKM de Cotonou

**Figure 2:**
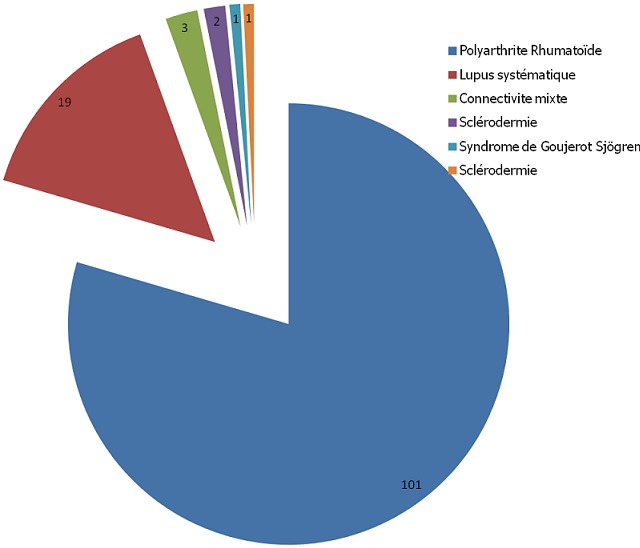
Répartition des connectivites ayant motivé la corticothérapie au long cours

Les pathologies ayant motivé la prescription de la corticothérapie étaient dominées par les névralgies cervico-brachiales (39%) et les connectivites (32%). La Figure 1 résume les différentes indications de la corticothérapie. 32% des patients ont présenté un tableau de connectivite ayant motivé la prescription d'une corticothérapie équivalente de 7,5mg/j de prednisone au moins au long cours (au-delà de 3 mois). La Figure 2 résume la répartition de ces différentes connectivites. 485 (65,1%) des patients avaient reçu une corticothérapie par voie générale. La prednisone et la prednisolone étaient les principales molécules utilisées pour la corticothérapie générale (environ 3/4 des cas) tandis que la bétaméthasone était la molécule la plus utilisée pour l'infiltration des différents sites articulaires. Le Tableau 1 résume les différents corticoïdes utilisés.

**Tableau 1:** Répartition des dérivés cortisoniques utilisés pour la corticothérapie générale

**Dérivés cortisoniques utilisés par voie générale**		
	**Nombre**	**Fréquence**
Prednisone	202	41,6
Prednisolone	168	34,6
Deflazacort	23	4,7
Betametasone	92	19,1
Total	485	100
**Dérivés cortisoniques utilisés lors des infiltrations**		
	Nombre	Fréquence
Betamethasone	97	37,3
Methylprednisone	76	29,2
Cortivasol	58	22,3
Triamcinolone	29	11,2
Total	260	100

### Les complications de la corticothérapie observées

Les complications étaient présentes chez 232 patients soit 31,1%. La corticothérapie générale était responsable de la prise de poids (dans plus d'un cas sur 2) et des crampes musculaires (38,4%). Les complications des infiltrations étaient dominées par la décoloration cutanée en regard du site d'injection (40%) et le hoquet (20%). Les Tableau 2 et Tableau 3 résument ces complications.

**Tableau 2:** Les complications engendrées par la corticothérapie générale

	Nombre (%)
Stimulation de l'appétit et prise de poids	104 (60,4)
Crampes musculaires aux membres pelviens	66 (38,4)
Epigastralgies	51 (29,6)
Irritabilité et troubles du sommeil	35 (20,3)
Hoquet	27 (15,7)
Constipation	21 (12,3)
Troubles trophiques cutanés	18(10,5))
OMI	6 (3,5)
Diabète	3 (1,7)
Autres*	13 (7,6)

* **HTA, hoquet, syndrome cushingoïde, gynécomastie, troubles menstruels, hirsutisme, dermatite**

**Tableau 3:** Effets secondaires engendrés par les infiltrations

	Nombre (%)
Décoloration cutanée	24 (40)
hoquet	12 (20)
Réaction de cristallisation	6(10)
Syndrome post-PL	4 (6,6)
Atrophie cutanée	1(1,7)
Diabète cortico-induit	1(1,7)
**Total**	60(100)

## Discussion

Les indications de la corticothérapie chez les sujets ouest-africains dans la pratique rhumatologique au Bénin sont multiples et représentaient 17,1% des prescriptions médicales. Les connectivites (notamment la polyarthrite rhumatoïde et le lupus érythémateux) occupaient une part importante et sont à l'origine d'une prescription de la corticothérapie dans environ plus de la moitié des cas. Nassar et coll. à Casblanca en 2014 a retrouvé un taux de 62,3% en ce qui concerne la prescription de la corticothérapie générale. L'indication majeure dans leur série était la polyarthrite rhumatoïde (50,4%) suivie du lupus et des vascularites [8]. De même, Lamchahab et coll. ont identifié dans leurs travaux que les principaux motifs de prescription de la corticothérapie, évoqués par les médecins, étaient les connectivites (91,1%) et les vascularites (26,3%) [9]. L’âge moyen des sujets était de 46,5 ans ce qui témoigne de la prédominance des affections rhumatologiques notamment les rhumatismes inflammatoires chroniques chez les sujets adultes jeunes. Les complications étaient observées dans près d'un cas sur trois. La fréquence de la plupart des effets indésirables attribués à une corticothérapie systémique a rarement été évaluée de façon systématique [2, 6]. Dans une étude prospective française portant sur 80 patients, Fardet et coll. [7] ont montré que 71% des patients rapportent au moins un effet indésirable et 66% au moins un effet indésirable qualifié de pénible. Le faible taux observé dans notre série pourrait être lié à la négligence de certains effets qualifiés de « minimes » par le patient et ou par le médecin. Par ailleurs, l'effet bénéfique presque spectaculaire de la corticothérapie dans certaines indications chez nos patients peut renvoyer au second plan voire vers la négligence des effets secondaires. La prise de poids, les crampes musculaires, le hoquet et les épigastralgies étaient les principaux effets indésirables de la corticothérapie générale signalés par nos patients.

Ces données sont globalement conformes aux données publiées en 2009 et 2014 respectivement par Perdoncini-Roux et coll. et Nassar et coll. En effet pour les premiers, La prise de poids était présente dans plus de 2/3 des cas suivis des complications cutanées dans plus d'un cas sur 4. Le déséquilibre tensionnel, le diabète, les complications neuropsychiatriques et l'ostéoporose sont des effets rencontrés dans leur série mais à un moindre degré [10]. Quant aux seconds auteurs, seules les complications métaboliques (prise de poids) et cutanées sont très fréquemment signalées (respectivement dans 59,4% et 23,2% des cas), l'ostéoporose était une complication rare dans leur série [8]. Cette rareté de l'ostéoporose dans leur série tout comme l'absence de cette complication dans notre série peut s'expliquer par l'absence de dépistage active de cette pathologie au Bénin liée à l'indisponibilité d'une ostéodensitométrie dans notre pays. En effet les travaux de Bouvard et al., ont révélé que l'ostéoporose était présente et asymptomatique chez 50% des patients exposés à la corticothérapie systémique[11]. Localement, les effets généraux signalés étaient dominés par le hoquet. Cet effet tout comme le flush ou les réactions d'hypersensibilité sont parfois dus à des conservateurs (sulfites) présents dans des préparations injectables. On pourrait aussi évoquer un passage systémique du produit cortisonique lors des infiltrations [12]. Des complications infectieuses ont été décrites: abcès épiduraux, méningites bactériennes et arthrites septiques interapophysaires postérieures. Elles sont très rares. Le syndrome post-ponction lombaire (céphalées, nausées et vomissements, raideur rachidienne) est signalé chez 10 à 30% des patients selon les séries [12]. L'administration de corticoïdes, fut-ce par voie locale, expose aussi aux effets secondaires de la corticothérapie par voie générale: freination de l'axe corticotrope, myopathie, rétention hydrosodée, décompensation d'un diabète sucré [12, 13].

## Conclusion

Les complications de la thérapie cortisonique sont diverses, variées et parfois gênantes conduisant à des inobservances thérapeutiques. La manipulation des dérivés cortisoniques doit se faire avec prudence en tenant compte du terrain et surtout de l’évaluation à chaque moment du rapport bénéfice-risque.
